# A clinical case of the penile metastasis from the rectal carcinoma

**DOI:** 10.2478/v10019-010-0004-1

**Published:** 2010-05-24

**Authors:** Mehmet Yildirim, Ali Coskun, Mete Pürten, Ozgur Oztekin, Enver Ilhan

**Affiliations:** Department of Surgery, Department of Radiology, Izmir Bozyaka Teaching and Research Hospital, Izmir, Turkey

**Keywords:** penis, metastasis, rectum, carcinoma

## Abstract

**Background:**

Penile metastases are rare and usually secondary to genitourinary and colorectal cancer.

**Case report:**

We present a case of a 77-year-old man with penile metastasis who was operated for rectal carcinoma. He was referred to our clinic for penile ulcerous lesion, semierectile penis and voiding dysfunction. Imaging studies showed nodular lesion at glans penis and multiple bone metastases. He did not respond to chemoradiotherapy and he had bad prognosis.

**Conclusions:**

Imaging methods and biopsy may help to clarify the diagnosis but the treatment modalities are insufficient in these patients.

## Introduction

The metastatic involvement of the penis is extremely rare, despite rich vascularisation between the penis and the neighbouring organs. The first report of secondary penile malignancy from an adenocarcinoma of the rectum was defined by Eberth.^1^ The commonest sites of primary malignancy are genito-urinary organs, rectum and recto-sigmoid areas.^2^ In literature less than 300 penile metastases were reported; 50 of them are originated from colorectal carcinoma.^3^ Penile metastases lead to semi-erectile penis/priapism and skin lesions.^2^ Like in colorectal lesions MRI is the most useful method for the accurate diagnosis in suspected penile metastases.^4^,^5^ An open biopsy is definitive for a the accurate diagnosis. A high rate of suspicion is required to detect them.

In this article, we report a case of a penile metastasis secondary to a rectal carcinoma three years after the surgery.

## Case report

A 78-year-old male presented in March 2002 with a 2 months history of change in bowel habit and rectal bleeding. The examination revealed a tumour of the lower rectum. An abdomino-perineal excision was performed and the pathological examination revealed a Dukes C undifferentiated adenocarcinoma with the lymph nodes involvement (15/22). He had received adjuvant chemo-radiotherapy postoperatively. He remained well until 2 years later, when he presented with urogenital complaints: penile ulcerous lesion, semierectile penis and voiding dysfunction.

**FIGURE 1:**
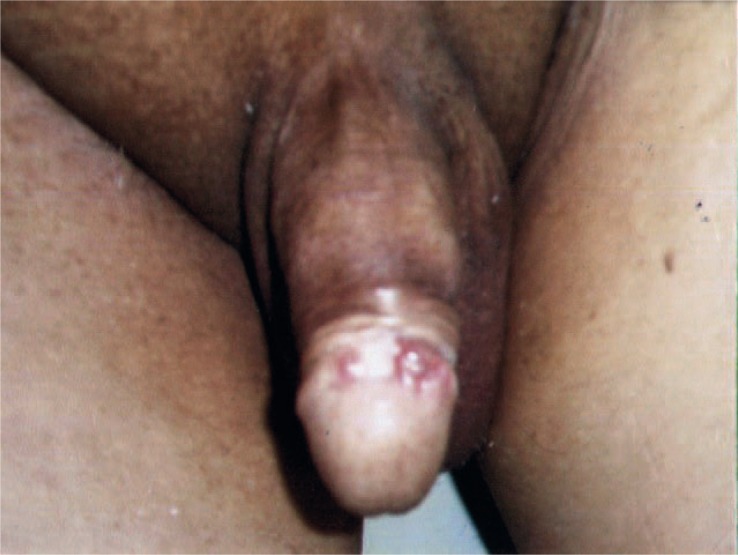
Physical examination showed indurations, oedema and ulcerative lesions of glans penis.

A follow-up examination was performed in April 2005 following the development of indurations, hyperaemia, and oedema of the penis and demonstrated 0.5 cm and 0.3 cm ulcerative lesions on glans penis (Figure 1). The examination of the superficial inguinal lymph nodes and the abdominal examination showed no remarkable signs. There was also no history of trauma or infectious disease.

**FIGURE 2:**
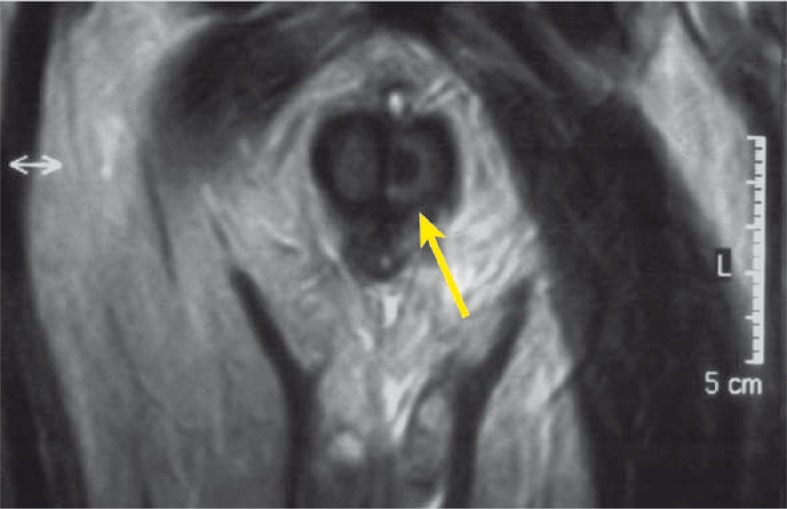
Axial T2-weighted MR image was showed nodular metastatic involvement of the penis shaft (arrow).

**FIGURE 3:**
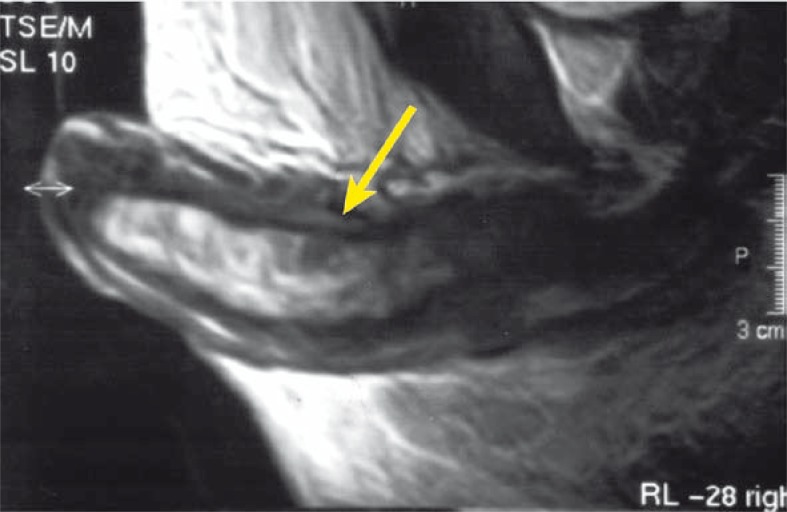
Sagittal T2-weighted MR image of the penis, demonstrating multiple metastatic nodules (arrow).

A complete blood cell count showed an increased white blood cell count of 16 x 10^3^ / μL (normal range: 5–10.0 x 10^3^/μL), anaemia (with a haemoglobin of 10.2 g/dL), normal level of PSA but increased levels of CEA (66.6 ng/ml, normal value <3 ng/ml) and CA19.9 (67.5 U/ml, normal value <37 U/ml). Penile US was normal for *corpus spongiosum* and *cavernosus*. CT demonstrated metastases of several thoracic vertebras and sacroiliac joint. Furthermore, MRI of the penis showed multiple pathological focuses in T2 signal and a nodular lesion that measured 15 mm in size, occupying the glans penis (Figures 2,3). The patient underwent excisional biopsy of the glans penis and inguinal lymph node, which revealed metastatic adenocarcinoma consistent with his rectal carcinoma. The patient received chemotherapy for metastatic disease until disease progression and unacceptable toxicity. The order of drug administration each week was irinotecan (80 mg/m^2^ i.v. day 1) followed by leucovorin (500 mg/m^2^ i.v day 1) and 5-fluorouracil (2300 mg/m^2^ continuous infusion day 1). Two days after chemotherapy administration of the third cycle the patient was admitted to our department with fatigue, weakness, dysuria and hypotension. Increased level of BUN, creatinin and decreased level of blood count were registered. Despite the whole blood transfusion, the values of blood count decreased (down to 5.4 g/L) during hospitalization. He was admitted for further medical therapy but during this admission his general condition deteriorated and he died with progressive disease.

Overall it appeared that this patient had a disseminated metastatic rectal carcinoma, with an unusual settled for the penis.

## Discussion

The penile metastasis in the course of rectal cancer is an unexpected complication, despite its proximity to the rectum and its rich vascularity. Recently published studies showed that the penile metastases are associated with prostate, urinary bladder cancer, and infrequently with rectal carcinoma.^6^ There have also been some reports regarding disseminated metastases from the oeasophagus, pancreas and stomach carcinoma. There are a few pathways of metastasis to penis; frequently, retrograde venous route, embolism to arterial system, retrograde lymphatic spread into the penile lymphatic channels, direct extension, and operative manuplations. The retrograde venous transportation suggested the main pathways to penis metastasis.^1^

The most common symptoms and signs are dysuria, voiding dysfunction, perineal pain, priapism, penile nodules and mass.^6^ In our case we found voiding dysfunction, semi-erectile penis and ulcerative lesions of the glans penis. Perineal pain, which it was a feature in one-third of patients in the series^5^, was not found in our case. We found a three-year interval between the primary tumour and penile metastasis. However, there are reports of the metastatic involvement years after the rectum carcinoma, but, penile metastases were usually reported on the average of 13 months after the primary carcinoma.^1^ The lung, vertebra and liver metastasis can be associated with the penile metastasis in patients with rectal carcinoma^2^ which, in our case, we found vertebral metastases at presentation. This might reflect the fact that penile metastases reflect the disseminated disease.

We believe that it would be beneficial in the differential diagnosis to take into consideration the findings related to voiding dysfunction, priapism and ulcerous lesions of the penis. Peyronie’s disease, traumatic and infectious skin and syphilitic lesions or primary carcinoma of penis, must also be in the differential diagnosis of the penile metastasis.^2^,^7^

Tissue tumour markers: AFP, CEA, HCG can be used for differential diagnosis. Penile US and CT is the first choice for imaging but its sensitivity is limited. Cavernosogram was reported a preferable application in some patients.^8^ MRI reported as a very useful method even at the beginning of the symptoms.^5^ MRI demonstrates multiple metastatic nodules, with a low signal intensity and is intense with the surrounding *corpus cavernosum* on T1-weighted images, and low signal intensity against the high background intensity of the cavernous bodies on T2-weighted imaging.^9^ Biopsy of the penile lesions is a favoured diagnostic modality to confirm the diagnosis as outlined in our case report. Regardless of the length to metastasis and difference in the treatment of the metastatic focus, the metastasis of the penis reflects a widely disseminated disease and poor prognosis.

Treatment options are palliative with local surgery, systemic chemotherapy and local radiotherapy.^4^,^7^ Surgery (panectomy) remains the only treatment with a long survival but mostly a patient dies in a year. Radiotherapy has an average survival of 8 months, whereas chemotherapy has not been studied for metastases from the rectum carcinoma. In our case, metastases did not respond to palliative therapy and showed progression of the local disease. Despite the studies showed that rectal primaries had longer surviving comparing to genitor-urinary primary, we did not find any forms of result.^7^,^8^,^10^

In conclusion, penile metastases are rarely seen as a form of metastasis among rectal cancer patients. Imaging methods may help to clarify the diagnosis, but penile lesions should be biopsied to confirm the diagnosis. On the other hand, the treatment modalities are insufficient for a long survey and quality of life in these patients.
